# Pore-Scale Mechanisms and Enhanced Oil Recovery Performance of Polymer-Assisted Winsor Microemulsion Systems: From Single Systems to Optimized Slug Design

**DOI:** 10.3390/polym18111361

**Published:** 2026-05-30

**Authors:** Xiaoqin Zhang, Feng Pan, Ibrahim I. Ramatou, Yongwang Liu, Xuan Zhou, Yiqiang Li, Bo Li, Kun Gao, Zheyu Liu

**Affiliations:** 1 National Energy Research and Development Center for Continuous Exploitation of Continental Sandstone Old Oilfields, Daqing 163712, China; zhangxiaoqin@petrochina.com.cn (X.Z.); panfeng1985@petrochina.com.cn (F.P.); zhouxuandq@petrochina.com.cn (X.Z.); libo123@petrochina.com.cn (B.L.); gaokun10@petrochina.com.cn (K.G.); 2 Exploration and Development Research Institute of Daqing Oilfield Co., Ltd., Daqing 163712, China; 3 College of Petroleum Engineering, China University of Petroleum (Beijing), Beijing 102249, China; ribrahimmaly@gmail.com (I.I.R.); yiqiangli@cup.edu.cn (Y.L.)

**Keywords:** Winsor phase, microemulsion flooding, pore-scale transport, residual oil mobilization, slug design

## Abstract

Polymer flooding is a highly promising enhanced oil recovery (EOR) technique for improving sweep efficiency, particularly in complex reservoirs at advanced stages of water production. While polymer flooding effectively improves sweep efficiency, efficient mobilization of residual oil requires further reduction in interfacial tension. Surfactant systems capable of forming microemulsions have therefore been introduced to enhance oil displacement through improved oil mobilization. The underlying oil displacement mechanisms of microemulsions are strongly dependent on phase behavior, which is governed by Winsor phase conditions. In this study, the pore-scale oil displacement mechanisms of Winsor I, II, and III microemulsion systems were systematically investigated using glass micromodel experiments. Winsor I mainly promoted oil detachment and emulsification, leaving residual oil as corner-bound oil and dispersed droplets. Winsor II showed limited efficiency due to its oil-continuous nature and viscous water-in-oil emulsions, resulting in persistent columnar residual oil. In contrast, Winsor III formed a continuous middle-phase microemulsion, enabling a solubilization-migration mechanism that effectively mobilized and transported oil. Accordingly, Winsor III achieved the highest recovery (81.37%), followed by Winsor I (75.6%) and Winsor II (64.9%). Optimized microemulsion slug injection further improved performance, with Winsor II-III-I reaching 82.2% and Winsor III-I sequence achieved the highest recovery of 85.6%. This study provides a mechanistic framework linking Winsor phase behavior to oil mobilization and demonstrates that both phase optimization and slug design are critical for improving microemulsion flooding performance in complex reservoir conditions.

## 1. Introduction

Polymer flooding is one of the most widely applied chemical enhanced oil recovery (EOR) techniques and has demonstrated significant success in improving oil recovery in mature reservoirs. Its effectiveness mainly arises from its ability to modify the flow behavior of the injected water by increasing its viscosity and reducing the relative permeability of the aqueous phase. As a result, the mobility ratio between the displacing and displaced fluids is improved, leading to better sweep efficiency and a more uniform displacement front [1,2,3,4,5,6,7]. Beyond macroscopic sweep improvement, polymer flooding can also contribute at the pore scale by mobilizing trapped oil through several mechanisms, including deformation and elongation of oil ganglia, detachment of oil from pore surfaces, and enhanced displacement of oil threads under flow [8,9,10,11]. Due to these advantages, polymer flooding has been extensively implemented in major oilfields where it has delivered notable increases in oil recovery [12,13]. However, despite its effectiveness in improving sweep efficiency, polymer flooding alone remains limited in its ability to mobilize trapped residual oil, particularly under conditions dominated by capillary forces and complex pore structures.

With the progressive depletion of conventional oil reserves, a large amount of residual oil remains trapped in reservoirs due to capillary forces and reservoir heterogeneity [14,15]. In situ emulsification was one of the first mechanisms investigated in chemical flooding studies and may initially occur during the contact between the injected surfactant system and crude oil within the porous media. Previous studies have shown that this process can contribute to enhanced oil recovery by improving residual oil mobilization and sweep efficiency [16]. Microemulsion flooding has been widely recognized as an effective method to improve oil recovery by reducing interfacial tension (IFT) and enhancing oil mobilization [17,18,19,20,21,22,23,24,25,26,27]. Microemulsions formed by surfactant systems can significantly lower the oil–water interfacial tension to ultra-low levels, thereby overcoming capillary trapping and improving displacement efficiency. Daqing Oilfield has long been a leading example of large-scale chemical flooding application, particularly through alkali–surfactant–polymer (ASP) flooding, which has achieved significant improvements in oil recovery [28]. Recently, attention has shifted toward polymer microemulsion-based systems, especially alkali-free formulations, as a promising alternative for improving performance in more complex reservoirs.

Microemulsion phase behavior is commonly classified according to the Winsor system (1948), which describes the equilibrium distribution of oil, water, and surfactant phases. In a Winsor I system, the microemulsion is water-continuous (oil-in-water), coexisting with excess oil, whereas in a Winsor II system, the microemulsion is oil-continuous (water in oil), coexisting with excess water. The Winsor III system is characterized by the formation of a middle-phase microemulsion in equilibrium with excess oil and water, representing a balanced state of surfactant affinity [29].

The performance of microemulsion flooding is strongly dependent on phase behavior. The Winsor III system, characterized by the formation of a middle-phase microemulsion, is generally considered the most favorable for oil recovery due to its balanced solubilization of oil and water and its ability to achieve ultra-low IFT [30,31]. Furthermore, different Winsor phases exhibit fundamentally distinct physicochemical characteristics. Winsor I systems are water-continuous with limited oil solubilization, Winsor II systems are oil-continuous with poor interaction with aqueous phases, and Winsor III systems represent a balanced middle phase capable of achieving ultra-low interfacial tension and high solubilization capacity. However, how these differences translate into oil displacement mechanisms in porous media remains unclear. Despite extensive studies on phase behavior and interfacial properties, the pore-scale mechanisms governing oil displacement under different Winsor conditions are not fully understood, particularly when the polymer is incorporated to improve mobility control.

Recent studies have shown that polymer-assisted microemulsion systems can enhance sweep efficiency and stabilize displacement fronts [32,33,34,35,36,37]. However, the coupling effect between the polymer, phase behavior, and pore-scale oil mobilization mechanisms is still unclear. Most existing studies focus on macroscopic recovery or bulk phase behavior, while limited attention has been given to the direct visualization of oil displacement processes and residual oil evolution within porous media. Understanding how different microemulsion systems interact with trapped oil at the pore scale is essential for optimizing chemical formulations and improving EOR performance. Furthermore, previous studies have widely employed in situ visualization techniques, such as glass micromodel experiments, to directly observe fluid flow and oil displacement processes at the pore scale [36,38,39,40,41,42,43,44]. These approaches provide valuable insights into droplet formation, coalescence, deformation, and the evolution of residual oil morphology during chemical flooding. However, most of these studies focused on single systems or macroscopic trends, with limited attention to the comparative analysis of displacement mechanisms under different Winsor phase conditions, particularly in polymer-assisted microemulsion systems.

In this study, the pore-scale oil displacement mechanisms of polymer-assisted Winsor I, II, and III microemulsion systems were systematically investigated using glass micromodel experiments. The microemulsion systems were formulated by adjusting salinity, and their interfacial properties and solubilization behavior were evaluated. By combining phase behavior analysis, interfacial tension measurements, and direct visualization of displacement processes, this work aims to clarify the relationship between Winsor phase behavior and oil mobilization mechanisms. In addition, the effect of optimized microemulsion slug injection strategies on oil recovery was explored. The findings provide new insights into the design of efficient microemulsion flooding systems for enhanced oil recovery.

Compared with previous pore-scale visualization studies, the present work does not only qualitatively observe residual oil mobilization, but systematically combines phase behavior analysis, interfacial tension characterization, pore-scale displacement visualization, pressure response, and core flooding performance to establish the coupling relationship between polymer-assisted mobility control and Winsor phase behavior during microemulsion flooding. In particular, this study emphasizes the distinct displacement mechanisms associated with Winsor I, Winsor II, and Winsor III systems and clarifies how polymer addition influences displacement stability, droplet transport, and oil mobilization efficiency.

## 2. Experimental Procedure

### 2.1. Materials

Surfactant, crude oil, brine, polymer, and porous media used in this study were primarily sourced from the Daqing Oilfield (Daqing, China).

**Figure 1:**
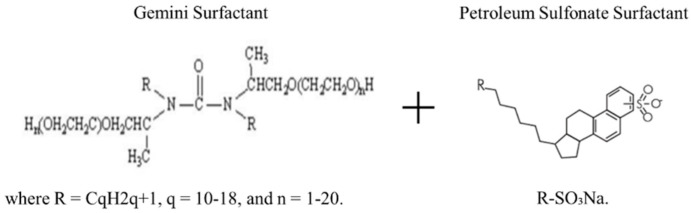
Chemical structures of the Gemini-type surfactant and Petroleum sulfonate constituting the mixed surfactant system used for microemulsion flooding.

The surfactant system used in this study consisted of a mixed surfactant formulation containing a Gemini-type surfactant and Petroleum sulfonate. The Gemini surfactant was based on Polyoxyethylene/Polyoxypropylene ether urea structures, while Petroleum sulfonate acted as the anionic component (Figure 1). The mixed surfactant system was used to improve phase behavior and interfacial activity during microemulsion flooding. The preferred mass ratio between Gemini surfactant and Petroleum sulfonate was approximately 6:4.

Crude oil composition is detailed in Table 1, and formation brine was also obtained from the same source, with the brine salinity detailed in Table 2. Polymer used in this study was partially hydrolyzed polyacrylamide (HPAM) with an average molecular weight of 1.9 × 10^7^ g/mol, supplied by Daqing Oilfield. Rock samples were representative of a Daqing A-type reservoir, with permeability ranging from 100 to 200 mD. These cores were used for core flooding experiments, while glass micromodels were employed for pore-scale visualization studies to simulate porous media conditions. Analytical-grade sodium chloride (NaCl) and sodium hydroxide (NaOH) were purchased from Tianjin Fuchen Chemical Reagent Co., Ltd. (Tianjin, China) and used as received without further purification.

### 2.2. Phase Behavior and Microemulsion Preparation

Microemulsions were prepared by systematically adjusting the salinity to obtain Winsor I, II, and III phase behaviors. The surfactant system consisted of a mixture of nonionic surfactant, with a total concentration fixed at 0.3 wt%. Aqueous solutions were prepared using a mixed electrolyte of NaCl and NaOH at a fixed mass ratio of 11:1, while the overall salinity was varied from 0.25 wt% to 2.5 wt% to identify the optimal phase transition conditions. The NaCl/NaOH ratio of 11:1 was selected based on preliminary phase behavior screening and previous alkaline–surfactant studies. NaCl mainly regulated the system salinity and promoted microemulsion phase transition, while the addition of a small amount of NaOH helped reduce interfacial tension through interfacial activity enhancement. Under the present experimental conditions, this ratio provided the most favorable phase behavior and interfacial properties. Equal volumes of crude oil and surfactant solution, typically 1:1, were introduced into sealed glass tubes, thoroughly mixed by repeated inversion, and then equilibrated at 45 °C for 24 h to ensure complete phase separation. The phase behavior was determined through visual observation, including the identification of phase volumes, interface clarity, and the presence of middle-phase microemulsion. Winsor types were classified based on the distribution of oil, water, and microemulsion phases at equilibrium.

### 2.3. Interfacial Tension Measurement

Interfacial tension (IFT) between crude oil and the prepared surfactant solutions was measured using a spinning drop tensiometer at 45 °C to evaluate the ability of each formulation to reduce capillary forces. The aqueous phase was the surfactant concentration 0.3 wt%, with salinity adjusted from 0.25 wt% to 2.5 wt% using a NaCl/NaOH solution. A droplet of crude oil was injected into a rotating capillary tube filled with the surfactant solution, and the rotational speed was gradually increased until a stable elongated oil droplet was formed. The IFT values were calculated based on the droplet shape and diameter under equilibrium conditions using the Vonnegut equation. Each measurement was repeated to ensure reproducibility, and the average value was reported.

### 2.4. Micromodel Visualization

**Figure 2:**
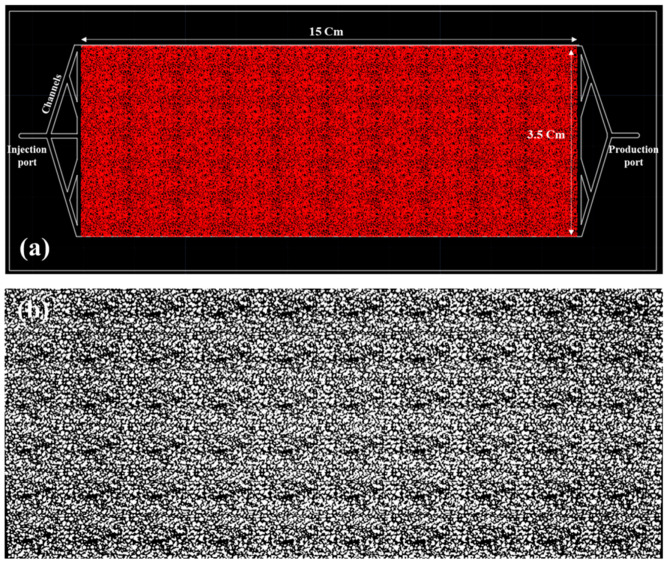
(**a**) Schematic of the glass micromodel; (**b**) segmented microscopic image.

The glass micromodel was fabricated using laser etching technology and consisted of a porous network designed to qualitatively simulate pore-scale flow behavior in sandstone reservoirs. The model dimensions were 15 cm × 3.5 cm, with pore-throat sizes ranging from approximately 10–50 μm and an estimated porosity of approximately 28%. The micromodel surface exhibited water-wet characteristics due to the glass material used for fabrication (Figure 2).

(1) The micromodel was first saturated with crude oil and subsequently water-flooded to establish residual oil conditions.
(2) Formation water was then injected at a constant flow rate until no further oil production was observed, establishing residual oil saturation within the micromodel.
(3) The prepared surfactant solutions corresponding to Winsor I, II, and III phase behaviors were then injected separately at a constant flow rate of 0.5 μL/min using a high-precision syringe pump. All experiments were performed at 45 °C to maintain consistency with phase behavior and interfacial tension measurements.
(4) The displacement processes were recorded using an optical microscopy system coupled with a high-resolution camera, allowing real-time observation of droplet dynamics and flow patterns. Images and videos were recorded continuously and later analyzed to evaluate oil displacement efficiency and pore-scale mechanisms for each Winsor system.
(5) These experiments enabled a direct comparison of the oil displacement mechanisms associated with different Winsor phase conditions at the pore scale.

### 2.5. Core Flooding Experiments

Core flooding experiments were conducted to evaluate oil recovery performance and to correlate pore-scale displacement mechanisms with macroscopic efficiency. Natural sandstone cores with a length of 10 cm and a diameter of 2.5 cm, with permeability varying from 100 to 200 mD and average porosity of 28%, were first vacuum-saturated with formation brine, followed by crude oil injection to establish initial oil saturation. Water flooding was then performed at a constant flow rate 0.15 mL/min until no further oil production was observed, establishing residual oil conditions. Subsequently, surfactant solutions corresponding to Winsor I, II, and III phase behaviors were injected at 45 °C. The injection was maintained at a constant rate to ensure consistent displacement conditions. During the flooding process, differential pressure across the core and cumulative oil recovery were continuously recorded. The recovery factor was calculated as a function of injected pore volume, enabling direct comparison of displacement efficiency among different microemulsion systems.

## 3. Results and Discussion

### 3.1. Winsor Phase Behavior and Interfacial Properties

**Figure 3:**
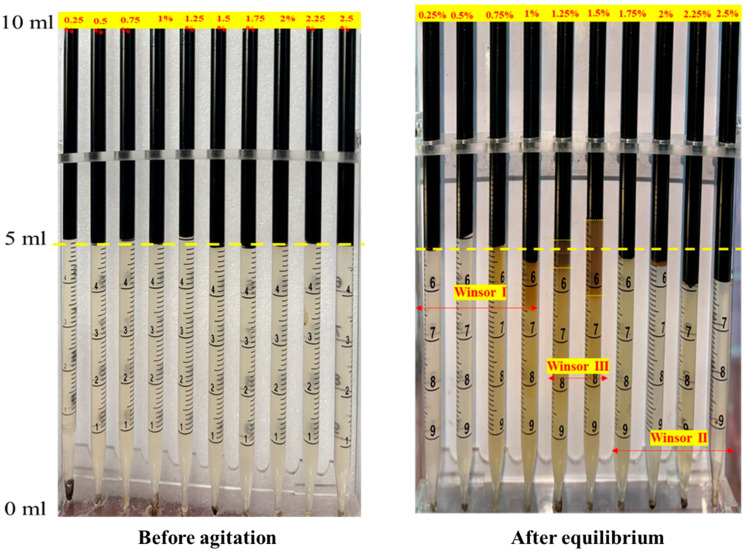
Salinity-dependent phase behavior of microemulsion system at 45 °C.

The phase behavior results show a clear transition from Winsor I to Winsor III and then to Winsor II as salinity increases (Figure 3).

At low salinity 0.25–0.75 wt%, the system is Winsor I, where the surfactant mainly stays in the water phase and oil solubilization is limited. As salinity increases to around 1.0–1.5 wt%, a middle-phase Winsor III microemulsion forms, indicating a balanced interaction between oil and water. This condition is typically associated with optimal solubilization and ultra-low interfacial tension. At higher salinity >1.75 wt%, the system shifts to Winsor II, where the surfactant prefers the oil phase and water is separated. This transition is mainly due to the effect of salinity, which reduces electrostatic repulsion and improves surfactant packing at the interface. The presence of NaOH may further enhance interfacial activity. The relatively wide Winsor III region suggests a good synergy between the nonionic surfactants, which is beneficial for enhanced oil recovery since it provides the most favorable conditions for oil mobilization.

**Figure 4:**
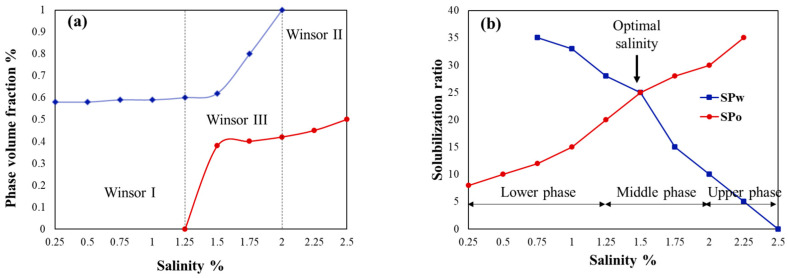
(**a**) Phase volume fraction of microemulsion system as a function of salinity at 45 °C; (**b**) variation in solubilization parameters (SPo and SPw) with salinity.

The solubilization parameters (SPo and SPw) were determined from the phase behavior tests by measuring the equilibrium volumes of oil and water solubilized in the microemulsion phase after 24 h of settling (Figure 4). SPo was calculated as the ratio of solubilized oil volume to surfactant volume, while SPw was defined as the ratio of solubilized water volume to surfactant volume. The results further confirm a clear phase transition in microemulsion behavior. As salinity increases, the system evolves from Winsor I to Winsor III and finally to Winsor II, which is consistent with the phase observations. This transition is well reflected in the solubilization parameters; SPo gradually increases while SPw decreases, indicating a shift in surfactant affinity from water to oil.

The intersection points of SPo and SPw at around 1.5 wt% define the optimal salinity, where balanced solubilization occurs and a stable middle-phase microemulsion is formed. This condition is particularly important, as it is typically associated with ultra-low interfacial tension and maximum oil mobilization. Overall, the agreement between phase behavior and solubilization trends highlights the reliability of the system and confirms that the Winsor III region provides the most favorable conditions for enhanced oil recovery.

**Figure 5:**
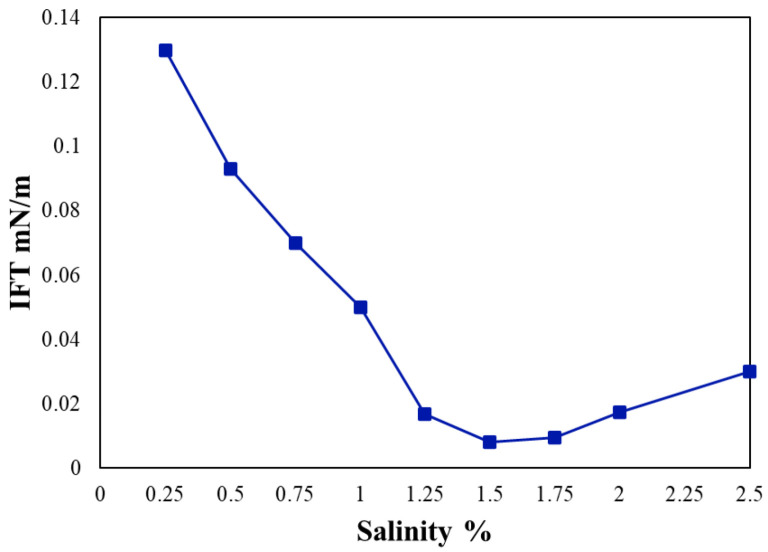
Variation in interfacial tension (IFT) between crude oil and the surfactant system as a function of salinity at 45 °C.

The interfacial tension (IFT) shows a strong dependence on salinity, decreasing from about 0.13 mN/m at 0.25 wt% to a minimum of approximately 0.008–0.01 mN/m around 1.5 wt% (Figure 5). This sharp reduction indicates that the surfactant system becomes much more effective at the oil–water interface, improving interfacial packing and activity. Beyond this point, the IFT gradually increases again, reaching about 0.03 mN/m at 2.5 wt%, suggesting that the surfactant starts to preferentially partition toward the oil phase. The minimum IFT near 1.5 wt% reaches 10^−3^ mN/m and indicates the optimal salinity identified from phase behavior and solubilization results, confirming the formation of a balanced Winsor III microemulsion. This consistency shows that the system is most effective at reducing capillary forces and enhancing oil mobilization near the optimal salinity.

### 3.2. Comparative Pore-Scale Oil Displacement Behavior of Winsor I, II, and III Systems

Microfluidic displacement experiments were conducted to compare the oil recovery mechanisms of different microemulsion systems under controlled conditions. The flooding sequence consisted of initial water flooding to establish residual oil saturation, followed by injection of a polymer-assisted microemulsion. The microemulsion systems were prepared at salinities corresponding to Winsor I (0.5 wt%), Winsor III (1.5 wt%), and Winsor II (2.0 wt%) conditions, with a fixed polymer concentration of 2150 mg/L. All injections were performed at a constant flow rate of 0.5 μL/min. This approach allows a direct comparison of how phase behavior influences displacement efficiency and pore-scale oil mobilization mechanisms.

#### 3.2.1. Pore-Scale Oil Displacement of Winsor I

**Figure 6:**
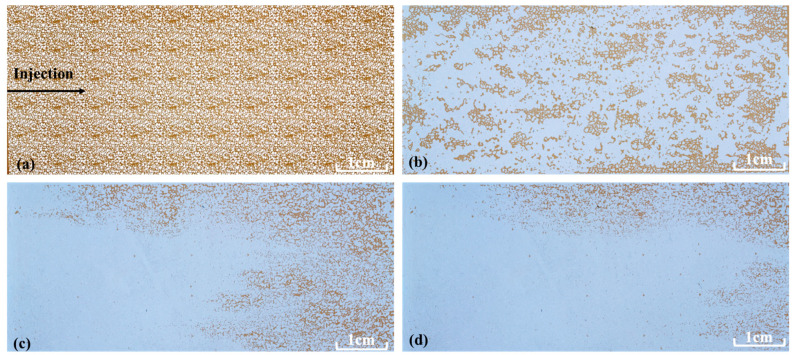
Pore-scale oil displacement process of Winsor I system: (**a**) oil-saturated state; (**b**) after water flooding; (**c**) after 0.5 PV microemulsion injection; (**d**) final stage of Winsor I flooding.

For the Winsor I system, the displacement images show a gradual but incomplete removal of residual oil (Figure 6). After initial oil saturation, water flooding leaves a significant amount of dispersed and significant residual oil in the pore network mainly trapped by capillary forces and pore-throat heterogeneity. After injecting 0.5 PV of Winsor I, part of the trapped oil is mobilized, and the swept region becomes clearer, indicating that the system can reduce oil–water interfacial tension and promote oil detachment from the pore walls. However, some oil clusters remain, especially in less-swept zones, suggesting limited solubilization capacity under Winsor I conditions. At the end of Winsor I injection, oil saturation is further reduced, but residual oil still appears as small droplets and isolated patches. This indicates that the main displacement mechanism is oil detachment, emulsification, and partial dispersion, assisted by the polymer-improved viscosity and mobility control. However, because Winsor I is water-continuous and has weaker oil solubilization than Winsor III, its ability to completely mobilize trapped oil is limited. Overall, the polymer-assisted Winsor I system improves sweep efficiency and removes part of the residual oil, but the displacement remains less efficient because oil mobilization mainly occurs through emulsification rather than strong middle-phase solubilization.

#### 3.2.2. Pore-Scale Oil Displacement of Winsor III

**Figure 7:**
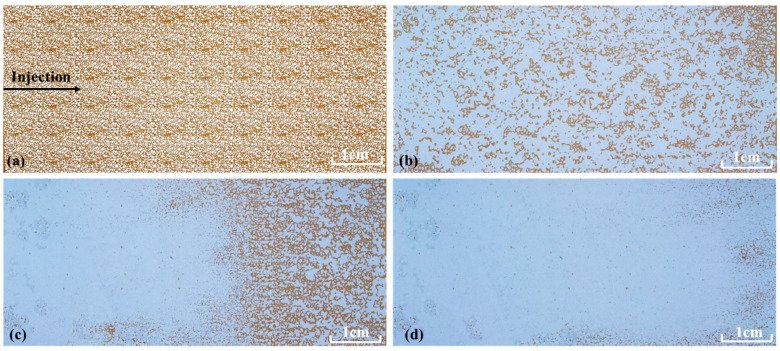
Pore-scale oil displacement process of Winsor III system: (**a**) oil-saturated state; (**b**) after water flooding; (**c**) after 0.5 PV microemulsion injection; (**d**) final stage of Winsor III flooding.

For the Winsor III system, the displacement process shows much more efficient and uniform oil removal compared to Winsor I (Figure 7). After water flooding, a large amount of residual oil remains. Upon injecting 0.5 PV of Winsor III microemulsion, a clear and continuous swept zone develops with a uniform displacement front, and rapid disappearance of oil clusters. This behavior indicates strong oil mobilization driven by the formation of a middle-phase microemulsion, which enables simultaneous solubilization of oil and water. The ultra-low interfacial tension significantly reduces capillary forces, allowing trapped oil to deform, reconnect, and flow through pore throats. In addition, the microemulsion facilitates a solubilization-migration mechanism, where oil is incorporated into the middle phase and transported out of the pore network. By the end of injection, the micromodel appears almost completely clean, with only minimal residual oil remaining as very fine droplets. This demonstrates that the dominant mechanism in Winsor III is not simple emulsification, but efficient mobilization and transport of oil through a balanced interfacial system. The presence of the polymer further enhances sweep efficiency by improving mobility control and stabilizing the displacement front. Overall, the Winsor III system provides the most effective displacement, combining ultra-low IFT, high solubilization capacity, and improved flow control, leading to superior oil recovery performance.

#### 3.2.3. Pore-Scale Oil Displacement of Winsor II

**Figure 8:**
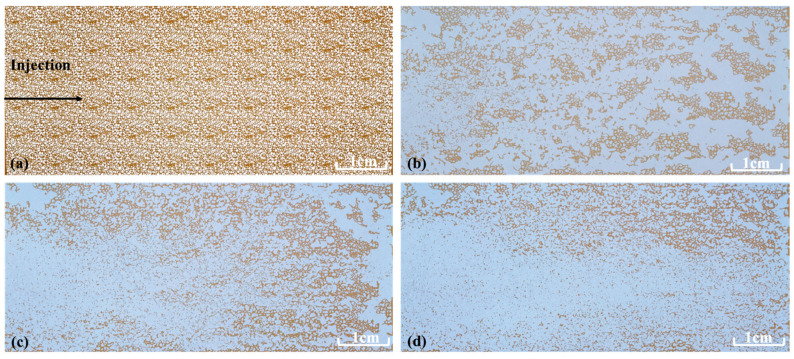
Pore-scale oil displacement process of Winsor II system: (**a**) oil-saturated state; (**b**) after water flooding; (**c**) after 0.5 PV microemulsion injection; (**d**) final stage of Winsor II flooding.

For the Winsor II system, the displacement behavior is noticeably less efficient compared to Winsor III and Winsor I (Figure 8). During Winsor II injection, some oil is mobilized, and partial sweeping is observed. However, large oil clusters persist, especially in unswept and low-flow regions. This indicates that although the system can reduce interfacial tension to some extent, its oil-continuous nature limits effective contact between the displacing phase and trapped oil. As injection proceeds, the overall oil saturation decreases slightly, but a considerable amount of residual oil remains as connected clusters and dispersed droplets. The dominant mechanism in this case is mainly limited mobilization and coalescence, rather than efficient solubilization. Because the microemulsion phase is oil-continuous, it tends to favor oil aggregation rather than breaking and transporting oil through the pore network. Although the presence of the polymer improves mobility control and sweep to some extent, it cannot compensate for the unfavorable phase behavior. As a result, the Winsor II system shows weaker displacement performance, with lower efficiency in removing trapped oil compared to the Winsor III system.

**Figure 9:**
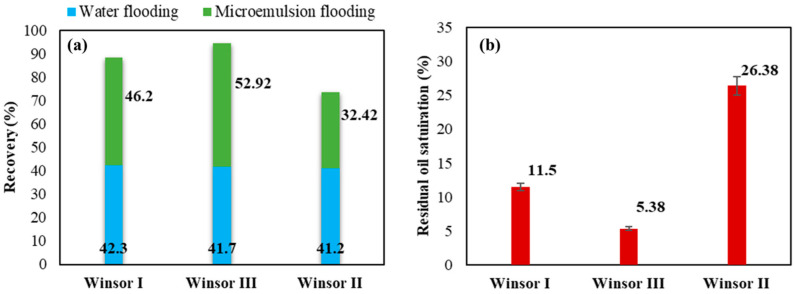
Comparison of recovery performance (**a**) and residual oil saturation (**b**) under different Winsor phase conditions during micromodel displacement experiments.

Image analysis was performed using Image J 1.53a software to quantitatively evaluate the displacement performance of different Winsor systems. The captured pore-scale images were converted into grayscale and binarized to distinguish residual oil from the swept regions. The residual oil area fraction was then calculated as the ratio of residual oil area to the total pore area after flooding. Figure 9 presents the recovery performance obtained from the micromodel displacement experiments under different Winsor phase conditions. During the initial water flooding stage, all systems exhibited similar recovery factors of approximately 41%, indicating that a considerable amount of residual oil remained trapped within the pore network after water flooding. Subsequent microemulsion flooding provided additional recovery after water flooding, with Winsor III showing the highest incremental recovery (52.92%), followed by Winsor I (46.2%) and Winsor II (32.42%). Consequently, the Winsor III system achieved the highest final recovery factor and the lowest residual oil saturation. The relatively high additional recovery obtained with Winsor I suggests that the system could still effectively mobilize part of the trapped residual oil through emulsification and interfacial tension reduction. However, compared with Winsor III, its displacement efficiency remained lower, likely due to less effective oil solubilization and transport behavior. In contrast, the poorer performance of the Winsor II system indicates that more residual oil remained trapped in the porous structure, possibly due to droplet accumulation and local flow resistance during displacement.

### 3.3. Residual Oil Morphology and Displacement Mechanism

**Figure 10:**
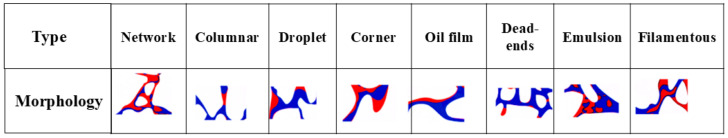
Residual oil morphology classification.

Residual oil morphology and classification were identified based on established criteria reported in previous pore-scale displacement studies [45]. Residual oil in porous media can be generally categorized into corner residual oil, film-like oil, columnar or connected oil, and dispersed droplets, depending on pore geometry, capillary forces, and displacement conditions (Figure 10).

#### 3.3.1. Residual Oil Morphology After Winsor I Flooding

**Figure 11:**
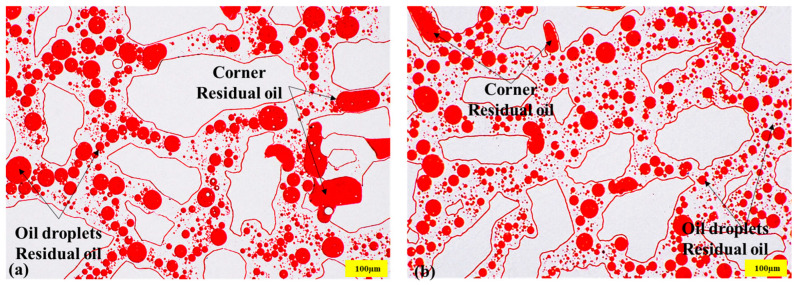
Residual oil morphology during Winsor I flooding: (**a**) early stage; (**b**) final stage.

After Winsor I flooding, a significant amount of residual oil remains distributed within the pore network, mainly in the form of corner residual oil and dispersed oil droplets. As shown in Figure 11, oil is preferentially trapped along pore corners and surface roughness due to capillary forces and wettability effects. Although the Winsor I system reduces interfacial tension to some extent, its water-continuous nature limits oil solubilization, resulting in incomplete mobilization of trapped oil. The displacement mechanism is therefore dominated by oil detachment and emulsification, where larger oil bodies are broken into smaller droplets. However, these droplets are not effectively transported and tend to remain trapped or re-accumulate in low-flow regions. The presence of the polymer improves sweep efficiency by stabilizing the displacement front, but it cannot fully overcome the limited solubilization capacity of the Winsor I system. Consequently, residual oil persists mainly as isolated droplets and corner-bound oil, indicating a relatively low displacement efficiency.

#### 3.3.2. Residual Oil Morphology After Winsor III Flooding

**Figure 12:**
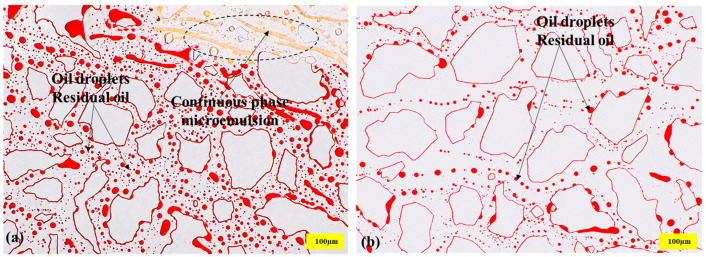
Residual oil morphology during Winsor III flooding: (**a**) early stage; (**b**) final stage.

After Winsor III flooding, the residual oil morphology is significantly altered compared to the Winsor I system. As shown in Figure 12, the formation of a continuous middle-phase microemulsion is clearly observed, which plays a key role in enhancing oil mobilization. Under these conditions, most of the residual oil is transformed into fine and uniformly dispersed oil droplets, indicating effective breakup of larger oil clusters. The ultra-low interfacial tension reduces capillary forces, allowing trapped oil to deform, detach, and be incorporated into the microemulsion phase. The dominant mechanism is therefore a solubilization-migration process, where oil is continuously solubilized into the middle phase and transported through the pore network. During subsequent water flooding, these small droplets are easily carried out due to their reduced size and weak attachment to pore surfaces. As a result, only minimal residual oil remains, mainly as very fine dispersed droplets, demonstrating a highly efficient displacement process. This confirms that the Winsor III system provides superior oil recovery by combining strong solubilization capacity with improved transport and sweep efficiency.

#### 3.3.3. Residual Oil Morphology After Winsor II Flooding

**Figure 13:**
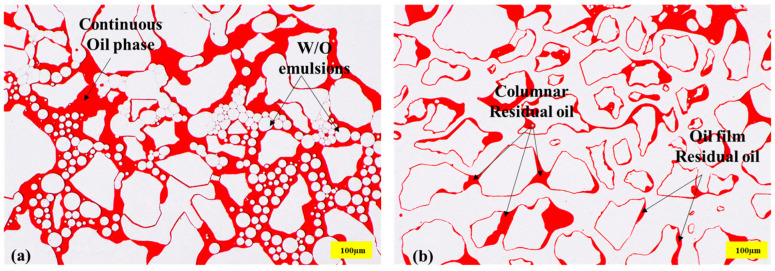
Residual oil morphology during Winsor II flooding: (**a**) early stage; (**b**) final stage.

At the early stage of Winsor II flooding (Figure 13), a water-in-oil (W/O) emulsion is formed, which increases the apparent viscosity of the displacing phase and reduces its mobility. Although the interfacial tension is significantly lowered, the oil-continuous nature of the Winsor II system limits effective contact between the injected fluid and trapped oil. As a result, the displacement is not fully efficient. The increased viscosity of the emulsion can locally improve sweep, but it also leads to flow resistance and non-uniform propagation of the displacement front. Mechanistically, oil mobilization occurs mainly through partial coalescence and deformation rather than efficient solubilization. Because the system lacks a stable middle-phase microemulsion, the solubilization-migration mechanism is weak, and oil tends to remain connected within pore channels. Consequently, the dominant residual oil form after Winsor II flooding is columnar and oil film residual oil, which remains trapped along flow paths due to insufficient breakup and transport. Overall, despite reduced IFT, the unfavorable phase structure and emulsion behavior limit the effectiveness of Winsor II in fully mobilizing residual oil.

Overall, a clear difference in residual oil morphology and displacement mechanisms is observed among the three systems. In the Winsor I system, residual oil mainly exists as corner-bound oil and dispersed droplets, reflecting a displacement dominated by oil detachment and emulsification. However, limited solubilization capacity restricts further mobilization, leading to moderate recovery. In contrast, the Winsor II system shows a less favorable behavior, where residual oil remains largely as connected and columnar structures, due to the oil-continuous nature of the phase and the formation of viscous water-in-oil emulsions. Although interfacial tension is reduced, oil mobilization is inefficient and transport is limited, resulting in the lowest displacement performance. On the other hand, the Winsor III system exhibits a fundamentally different mechanism, characterized by the formation of a continuous middle-phase microemulsion. This enables a solubilization-migration mechanism, where residual oil is effectively broken into fine droplets, solubilized, and transported through the pore network. Consequently, residual oil is minimal and mainly exists as small, isolated droplets that are easily removed during post-water flooding.

**Figure 14:**
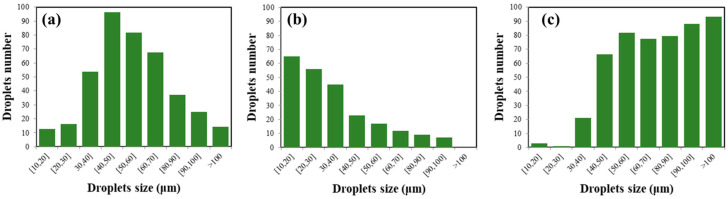
Analysis of droplet size distribution under different Winsor phase conditions during micromodel displacement experiments: (**a**) Winsor I; (**b**) Winsor III; (**c**) Winsor II.

Further quantitative analysis of oil droplets was conducted using image-processing techniques to better evaluate the displacement behavior under different Winsor phase conditions. Figure 14 shows the droplet size distribution under different Winsor phase conditions. The Winsor III system mainly generated droplets in the range of 10–50 μm, with fewer large droplets (>70 μm), indicating improved dispersion and transport stability during displacement. In contrast, the Winsor II system exhibited a significantly higher proportion of large droplets in the range of 70–100 μm and >100 μm, suggesting droplet coalescence and accumulation within pore channels, which may increase local flow resistance and reduce residual oil mobilization efficiency. The Winsor I system showed intermediate behavior, with most droplets distributed between 40 and 80 μm. These results further support the superior displacement performance of the Winsor III microemulsion system observed in the micromodel flooding experiments.

To provide a comprehensive comparison of the three systems, Table 3 summarizes the key differences in phase behavior, interfacial properties, displacement mechanisms, and oil recovery performance, highlighting the dominant role of the Winsor phase in governing displacement efficiency.

### 3.4. Macroscopic Oil Displacement Evaluation

**Figure 15:**
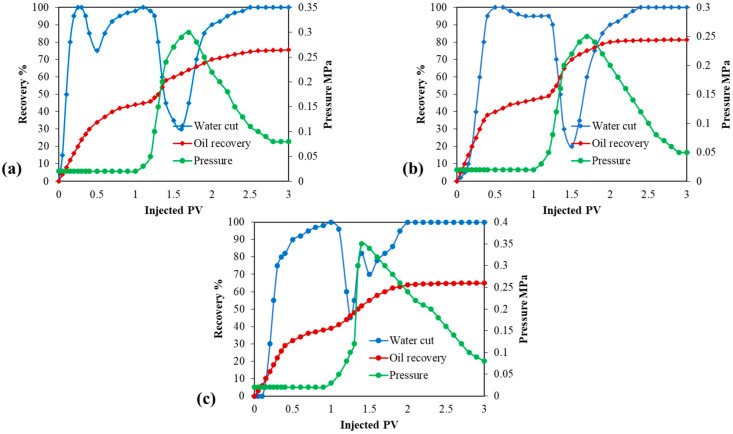
Comparison of oil recovery, water cut, and pressure profiles during microemulsion flooding for (**a**) Winsor I, (**b**) Winsor III, and (**c**) Winsor II systems.

The macroscopic oil displacement was evaluated through core flooding experiments. The results clearly demonstrate the influence of Winsor phase behavior on recovery performance under polymer-assisted microemulsion flooding (Figure 15). With a fixed injection of 0.3 PV microemulsion and 2150 mg/L polymer addition, the Winsor III system achieved the highest oil recovery of 81.37%, followed by Winsor I with 75.6%, while the Winsor II system exhibited the lowest recovery of 64.9%. In addition to phase behavior effects, the coupling relationship between polymer viscosity control and microemulsion transport also played an important role in the displacement performance. The enhanced displacement performance resulted from the coupling effect between polymer viscosity control and microemulsion phase behavior. The polymer increased the displacing-phase viscosity, thereby improving the mobility ratio and stabilizing the displacement front, which enhanced macroscopic sweep efficiency. Meanwhile, the microemulsion system reduced interfacial tension and promoted oil solubilization and mobilization at the pore scale. Under Winsor III conditions, the balanced phase behavior further improved droplet transport and residual oil displacement, resulting in superior recovery performance.

This enhanced recovery is attributed to the balanced phase structure of the Winsor III system, which generates ultra-low interfacial tension and enables an efficient solubilization-migration mechanism. This is further supported by the relatively moderate pressure peak of 0.24–0.26 MPa and a smooth reduction in water cut to 20–30%, indicating effective oil mobilization with limited flow resistance and stable transport through the pore network. In contrast, the Winsor I system, although capable of reducing interfacial tension, primarily promotes oil detachment and dispersion rather than continuous solubilization. As a result, recovery improves to a moderate level, but this is accompanied by a higher-pressure peak of 0.30–0.32 MPa and a water cut minimum of 30–40%, suggesting increased flow resistance and less efficient transport of mobilized oil. The relatively poor performance of the Winsor II system is evidenced by its lower recovery of 64.9%, despite exhibiting the highest-pressure peak of 0.34–0.36 MPa. This behavior indicates severe flow resistance caused by droplet coalescence and accumulation within pore throats. The oil-continuous nature of the Winsor II microemulsion reduces effective contact between the displacing phase and trapped oil, limiting solubilization and promoting blockage, as is also reflected by the fluctuating water cut behavior during injection. In contrast, the improved displacement stability observed for the Winsor III system was attributed to the combined effects of lower interfacial tension and enhanced mobility control. The addition of the polymer increased the displacing-phase viscosity, thereby improving the mobility ratio and stabilizing the displacement front. Meanwhile, the reduction in interfacial tension increased the capillary number, promoting the mobilization and transport of trapped residual oil within the porous media.

The comparison confirms that the Winsor III system provides the optimal balance between mobilization and transport, highlighting the critical importance of selecting appropriate phase conditions, particularly the formation of a stable Winsor III microemulsion for maximizing oil recovery in microemulsion flooding processes. Based on the above analysis, a slug injection strategy was designed to further evaluate the phase-dependent displacement mechanisms at the macroscopic scale.

### 3.5. Implications of Microemulsion Phase Slug Combination and Displacement Strategy

The slug combination experiments were designed to evaluate whether sequential Winsor phase injection could improve oil displacement by combining phase selective mobilization with polymer-assisted mobility control. In this study, two injection strategies were considered: Winsor II–Winsor III–Winsor I and Winsor III–Winsor I. The Winsor III-I sequence consisted of 0.3 PV Winsor III microemulsion followed by 0.2 PV Winsor I microemulsion. For the Winsor II-III-I sequence, 0.2 PV Winsor II microemulsion was first injected, followed by 0.3 PV Winsor III microemulsion and 0.2 PV Winsor I microemulsion. A polymer solution with a concentration of 2150 mg/L was further introduced to improve mobility control and stabilize the displacement front.

**Figure 16:**
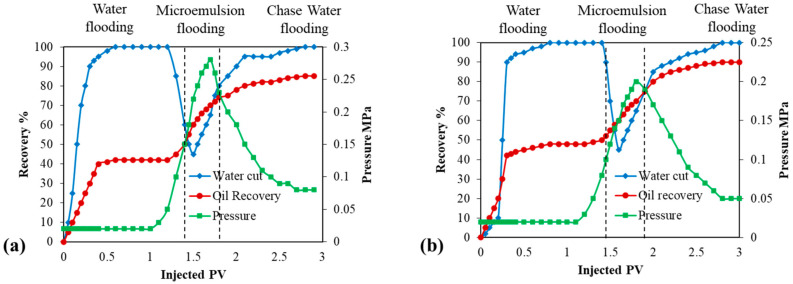
Comparison of oil recovery, water cut, and pressure profiles for (**a**) Winsor II-III-I and (**b**) Winsor III-I microemulsion slug injection strategies.

The recovery curves in Figure 16 clearly demonstrate the influence of slug design on oil displacement efficiency, pressure behavior, and flow dynamics. During the initial water flooding stage (0–1.5 PV), both cases exhibit similar trends, with water cut rapidly increasing to approximately 100% at around 0.5 PV, indicating early water breakthrough, while the pressure remains low and stable at 0.02–0.03 MPa, reflecting limited mobilization of residual oil under water flooding conditions. For the Winsor II-III-I sequence, oil recovery increases from 42% to 75% at 2.0 PV, and final recovery was 82.2%. The initial Winsor II slug may modify the oil-rich environment and promote contact between the surfactant system and residual oil. However, due to its oil-continuous nature and relatively weaker displacement efficiency, excessive Winsor II contribution may also increase the risk of droplet coalescence and pore-throat blockage. Therefore, its role is more likely related to phase conditioning rather than direct efficient oil mobilization. The subsequent Winsor III main slug plays the dominant role in residual oil mobilization. Owing to its ultra-low interfacial tension and balanced oil–water solubilization capacity, the Winsor III microemulsion promotes a solubilization-migration mechanism, allowing trapped oil to deform, dissolve, and migrate through the pore network. The use of 0.3 PV Winsor III as the main slug is therefore critical, as this phase provides the strongest contribution to capillary pressure reduction and microscopic oil displacement.

In the Winsor III-Winsor I strategy, oil recovery increases more efficiently from 48% to 80% at 2.0 PV and final recovery was 85.6%. The direct injection of Winsor III enables earlier formation of the most favorable displacement phase, enhancing microemulsion–oil contact from the beginning of the chemical slug stage. The following Winsor I slug, characterized by a water-continuous phase, may help disperse mobilized oil droplets and sustain forward transport. Compared with the three-step Winsor II-III-I sequence, this simplified strategy may reduce the risk of emulsion accumulation while maintaining the key advantage of Winsor III-driven solubilization. The addition of 2150 mg/L polymer further improves the displacement process by increasing aqueous-phase viscosity, reducing mobility contrast, and suppressing viscous fingering. This polymer-assisted mobility control is particularly important after microemulsion-induced oil mobilization, because the mobilized oil and dispersed droplets require a stable displacement front to be efficiently transported. Therefore, the enhanced recovery performance is not only controlled by Winsor phase behavior, but also by the coupling between Winsor III solubilization ability and polymer mobility control.

The pressure profiles provide additional insight into the displacement mechanisms. In case a, the pressure rises sharply to a peak of 0.28–0.30 MPa after introducing microemulsion, indicating significant flow resistance likely caused by droplet coalescence and pore-throat blockage associated with the initial Winsor II slug. In contrast, case b exhibits a lower pressure peak of 0.22–0.24 MPa, suggesting a more stable displacement process with reduced resistance and improved flow continuity. Overall, the results indicate that slug design should prioritize the early and effective placement of the Winsor III microemulsion, while using Winsor I and the polymer to support dispersion and mobility control. The comparison between Winsor II-III-I and Winsor III-I suggests that excessive reliance on Winsor II may be less favorable, whereas a Winsor III-I centered slug strategy combined with the polymer provides a more efficient route for residual oil mobilization in heterogeneous porous media.

To further evaluate the effectiveness of the proposed system, the recovery performance was compared with previously reported polymer–microemulsion flooding studies (Table 4). Compared with previously reported polymer–microemulsion flooding studies, the present work exhibited comparable or higher additional recovery after water flooding, particularly under Winsor III conditions. The improved performance was mainly associated with the coupling effect between favorable phase behavior, enhanced oil solubilization, and polymer-assisted mobility control.

Although the present study demonstrated promising laboratory-scale performance, further investigations on economic cost and reservoir-scale applicability are still required before field implementation of the polymer–microemulsion system.

## 4. Conclusions

This work demonstrates that the effectiveness of microemulsion flooding is governed not simply by interfacial tension reduction, but by the interplay between phase behavior and transport mechanisms at the pore scale. Although all systems reduced IFT, their displacement performance differed markedly due to the nature of the formed microemulsion. The Winsor I system improved oil detachment but remained limited by insufficient solubilization, while the Winsor II system suffered from unfavorable oil-continuous behavior and viscous emulsion formation, restricting flow and oil mobilization. In contrast, the Winsor III system provided a balanced interfacial environment that enabled continuous oil solubilization and transport, resulting in a more uniform sweep and minimal residual oil which resulted in the highest recovery among single systems (81.37%). Furthermore, the introduction of optimized microemulsion slug sequences significantly improved oil recovery. The Winsor II-III-I sequence achieved 82.2%, while the Winsor III-I sequence reached the highest recovery of 85.6%, demonstrating the advantage of combining different phase behaviors to enhance displacement efficiency.

These results indicate that beyond achieving optimal salinity, strategic slug design plays a critical role in maximizing oil recovery by integrating mobility control, interfacial activity, and transport mechanisms. Therefore, the design of chemical EOR systems should prioritize phase behavior optimization rather than relying solely on interfacial tension reduction. This study provides a pore-scale understanding that can guide the formulation of more effective microemulsion systems for field applications.

Future studies should further investigate the long-term transport behavior, surfactant retention, and reservoir-scale applicability of polymer-assisted Winsor microemulsion systems under heterogeneous reservoir conditions.

## Figures and Tables

**Table 1:** Crude oil composition.

Density (20 °C)	Kinematic Viscosity (50 °C)	Pour Point	Wax Content	Sulfur Content	Nitrogen Content	Resin Content	Asphaltene Content	Carbon Residue
0.86 g/cm^3^	20 mm^2^/s	30 °C	25 wt%	0.1–0.15 wt%	0.16 wt%	8.9 wt%	0–0.1 wt%	2.9–3.0 wt%

**Table 2:** Composition of formation brine.

Ion	Concentration (mg/L)
OH^−^	0.00
CO_3_^2−^	3.87 × 10^2^
HCO_3_^−^	4.52 × 10^3^
Cl^−^	1.29 × 10^3^
SO_4_^2−^	2.09 × 10^1^
Ca^2+^	4.37 × 10^1^
Mg^2+^	1.59 × 10^1^
Na^+^ + K^+^	2.77 × 10^3^
Total salinity	9.05 × 10^3^
pH	9.20

**Table 3:** Comparison of displacement mechanisms and residual oil characteristics under different Winsor systems.

Parameters	Winsor I	Winsor II	Winsor III
Phase type	Water-continuous	Oil-continuous	Middle-phase microemulsion
IFT behavior	Relatively low IFT	Relatively low IFT	Lowest IFT
Displacement mechanism	Detachment & emulsification	Limited mobilization & coalescence	Solubilization migration
Microemulsion structure	Oil in water	Water in oil	Continuous middle phase
Oil mobilization	Moderate	Low	High
Residual oil morphology	Corner oil & droplets	Columnar & connected oil	Fine dispersed droplets
Droplet size	Medium	Large and connected	Very small and uniform
Transport ability	Limited	Poor	Excellent

**Table 4:** Comparison of recovery performance between the present study and previously reported polymer–microemulsion flooding studies.

System	Flooding Type	Incremental Recovery (%)	Main Mechanism	References
Polymer-assisted Winsor III microemulsion	Microemulsion flooding	20.58% OOIP	Ultra-low IFT and oil solubilization	[32]
In situ surfactant micro-emulsification	Surfactant flooding	15–43% OOIP	In situ microemulsion formation and residual oil mobilization	[46,47]
Gemini surfactant/polymer flooding	Surfactant–polymer flooding	36.75% OOIP	Mobility control and slug injection	[48]
Present study	Polymer-assisted Winsor microemulsion flooding	32.42–52.92% additional recovery after water flooding	Coupling of phase behavior, oil solubilization, and mobility control	This work

## Data Availability

The data presented in this study are available on request from the corresponding author.
